# Impact of Age and Sex on Antibody Response Following the Second Dose of COVID-19 BNT162b2 mRNA Vaccine in Greek Healthcare Workers

**DOI:** 10.3390/microorganisms9081725

**Published:** 2021-08-13

**Authors:** Niki Vassilaki, Antonios N. Gargalionis, Anastasia Bletsa, Nikolaos Papamichalopoulos, Elisavet Kontou, Meropi Gkika, Kostas Patas, Dimitrios Theodoridis, Ioannis Manolis, Anastasios Ioannidis, Raphaela S. Milona, Alexandra Tsirogianni, Emmanouil Angelakis, Stylianos Chatzipanagiotou

**Affiliations:** 1 Laboratory of Molecular Virology, Hellenic Pasteur Institute, 127 Vasilissis Sofias Avenue, 11521 Athens, Greece; nikiv@pasteur.gr (N.V.); raphaelasmilona@gmail.com (R.S.M.); 2 Department of Medical Biopathology, Eginition Hospital, Athens Medical School, National and Kapodistrian University of Athens, 72–74 Vasilissis Sofias Avenue, 11528 Athens, Greece; agargalionis@yahoo.gr (A.N.G.); nikovasilis@yahoo.gr (N.P.); gikameri@yahoo.gr (M.G.); konpatas@gmail.com (K.P.); 3 Immunology-Histocompatibility Department of Evangelismos General Hospital, Ypsilantou Str. 45–47, 10676 Athens, Greece; anable_3@yahoo.com (A.B.); kontolisa@gmail.com (E.K.); alextsir@gmail.com (A.T.); 4 Department of Medical Biochemistry, Konstantopoulio-Patision General Hospital, Agias Olgas 3–5, 14233 Nea Ionia, Greece; dimdrteo@gmail.com (D.T.); manolisj@yahoo.gr (I.M.); 5 Department of Nursing, Faculty of Health Sciences, University of Peloponnese, Sehi Area, 22100 Tripoli, Greece; tasobi@gmail.com; 6 Department of Diagnostics, Hellenic Pasteur Institute, 127 Vasilissis Sofias Avenue, 11521 Athens, Greece; angelotasmanos@msn.com; 7 IRD, APHM, VITROME, IHU-Méditerranée Infection, Aix-Marseille University, 19–21 Boulevard Jean Moulin, 13005 Marseille, France

**Keywords:** COVID-19, BNT162b2 mRNA, SARS-CoV-2 IgG, healthcare workers

## Abstract

Anti-SARS-CoV-2 spike RBD (receptor-binding domain) IgG antibody levels were monitored in 1643 volunteer healthcare workers of Eginition, Evangelismos, and Konstantopoulio General Hospitals (Athens, Greece), who underwent vaccination with two doses of COVID-19 BNT162b2 mRNA vaccine (Pfizer) and had no history of SARS-CoV-2 infection. Venous blood was collected 20–30 days after the second vaccine dose and anti-RBD IgG levels were determined using CMIA SARS-CoV-2 IgG II Quant (Abbott) on ARCHITECT i System or ADVIA Centaur SARS-CoV-2 IgG (Siemens) on Centaur XP platform. From the total population of 1643 vaccinees (533 M/1110 F; median age = 49; interquartile range-IQR = 40–56), 1636 (99.6%) had anti-SARS-CoV-2 IgG titers above the positivity threshold of the assay used. One-Way ANOVA Kruskal-Wallis H test showed a statistically significant difference in the median of antibody titers between the different age groups (*p* < 0.0001). Consistently, Spearman’s correlation coefficient (r) for IgGs and age as continuous variables was −0.2380 (*p* = 1.98 × 10^−17^). Moreover, antibody titers were slightly higher by 1.2-mean fold (*p* = 3 × 10^−6^) in the total female population of the three hospitals (median = 1594; IQR = 875–2584) as compared to males (median = 1292; IQR = 671.9–2188). The present study supports that BNT162b2 vaccine is particularly effective in producing high anti-SARS-CoV-2 IgG levels in healthy individuals, and this humoral response is age- and gender-dependent.

## 1. Introduction

Severe acute respiratory syndrome coronavirus 2 (SARS-CoV-2) emerged as a pathogen in December 2019 and is responsible for coronavirus disease 2019 (COVID-19) [1]. This novel virus causes symptoms that range from fever, dry cough, and dyspnea to the development of acute pneumonia, while neurological complications have also been reported [2], including damage in the dopaminergic system [3,4]. SARS-CoV-2-infection can be fatal for patients with underlying conditions regardless of their age, while healthy individuals that are treated can recover within 2–4 weeks [5]. Until 31 July 2021, 4.22 million deaths due to coronavirus have been reported at ourworldindata.org. Shortly after the first detected cases, this outbreak was declared a pandemic by the World Health Organization (WHO), and the progress of the disease as well as the release of the pathogen’s full genomic sequence in public databases outlined and promoted the necessity of effective public health strategies such as the development of vaccines [6].

SARS-CoV-2 Spike protein, and specifically its receptor-binding domain (RBD), seems to be the dominant target of antibodies produced by infected patients [7]. Neutralization of this domain would interfere with its affinity to ACE2, an entry receptor in various human cells, and for this reason it presents the most promising target for a vaccination design. As a result, most vaccine candidates, including the Pfizer/BioNTech vaccine BNT162b2, aim at causing the production of RBD-binding antibodies [8]. This vaccine is a lipid nanoparticle (LNP)-formulated mRNA, which encodes a modified spike protein that is stable at a prefusion stage and stimulates a strong immune response [8,9]. It provokes a durable dose-dependent antibody production, regarding both RBD-binding IgG levels and neutralizing titers that show a strong correlation, and elicits T cell responses [10]. The vaccine BNT162b2 is FDA (Food and Drug Administration)-approved and has been shown to be 95% effective in phase III of clinical trials [11]. Interestingly, the antibodies caused by the vaccine show great structural similarity with those produced by natural infection [12]. Side effects can also occur, as in all vaccines, and include pain, fatigue, fever, and headaches, and in some cases, there have been reports of allergic reactions to some of the vaccine components [13,14]. In total, this technology allows for a low-cost, scalable production of vaccines in a short time, becoming one of the best solutions while facing a pandemic [15].

Some concerns have been raised about the Pfizer vaccine regarding the inter-individual homogeneity of the SARS-CoV-2 antibody levels it elicits in the general population. Studies have indicated that psychological, physiological, and behavioral factors could play a major role in the intensity of the immune response provoked by the vaccine [16,17,18]. Studies have consistently reported higher morbidity and mortality of SARS-CoV-2 infection in women and people of older age, excluding children younger than 5 years old that are also vulnerable [19,20,21]. However, concerning the impact of age and sex on vaccine efficiency, the data so far are limited and contradictory [18,22,23,24,25,26,27]. Thus, more experiments should be conducted to determine this relationship. Such information will help to identify any related specific populations that may be low vaccine responders. It is important to consider this while planning population health risk stratification strategies, especially since vaccine prioritization efforts are underway both nationally and globally.

This study aims to clarify whether there is a heterogeneity in the anti-SARS-CoV-2 spike IgG antibody titers produced by the Pfizer BNT162b2 vaccine among individuals with different ages and sexes, by examining the sera of 1643 health workers that have received both doses of the vaccine and have successfully produced antibodies against SARS-CoV-2, in routinely used diagnostic chemiluminescent immunoassays. The ages of the subjects of the study range from 23 to 93 years old and both female and male workers were included in the study, providing an adequate sample able to assist us in determining if the vaccine elicits an age- or sex-dependent immune response.

## 2. Materials and Methods

### 2.1. Sample Collection

Assessed samples comprised 1643 sera from volunteers vaccinated with the COVID-19 BNT162b2 vaccine (Comirnaty; BioNTech and Pfizer), a lipid nanoparticle-formulated, nucleoside-modified mRNA encoding the SARS-CoV-2 spike protein receptor binding domain (RBD). The vaccinees were healthcare workers at Eginition, Evangelismos, and Konstantopoulio General Hospitals (Athens, Greece). Persons over 67 years of age were retired former workers in healthcare, still serving in scientific or academic assignments. Inclusion criteria for participation in this study included: (a) age above 18 years; (b) ability to sign the informed consent form; (c) eligibility for vaccination, according to the national program for COVID-19 vaccination. Exclusion criteria included: (a) history or laboratory evidence of prior SARS-CoV-2 infection, (b) reception of immunosuppressive therapy, and (c) history of allergic reactions to drugs or vaccines. Two vaccine doses of 30 μg were prepared following the manufacturer’s instruction and administered with an interval of 21 days to the study participants, within 30 min from resuspension. Venous blood was collected 20–30 days after the second vaccine dose, into evacuated blood tubes containing gel and clot activator (Vacutainer, BD, Franklin Lakes, NJ, USA). Serum was separated by centrifugation (15 min, 1500× *g*, room temperature) and stored at 2 to 8 °C prior to assessment for a maximum of 2 days.

### 2.2. Ethics

Informed written consent was obtained from all participants in the study. The study was conducted according to the guidelines of the Declaration of Helsinki and approved by the Institutional Review Board of Evangelismos General Hospital (PN 9/21-01-21).

### 2.3. Serological Assays

**Figure 1:**
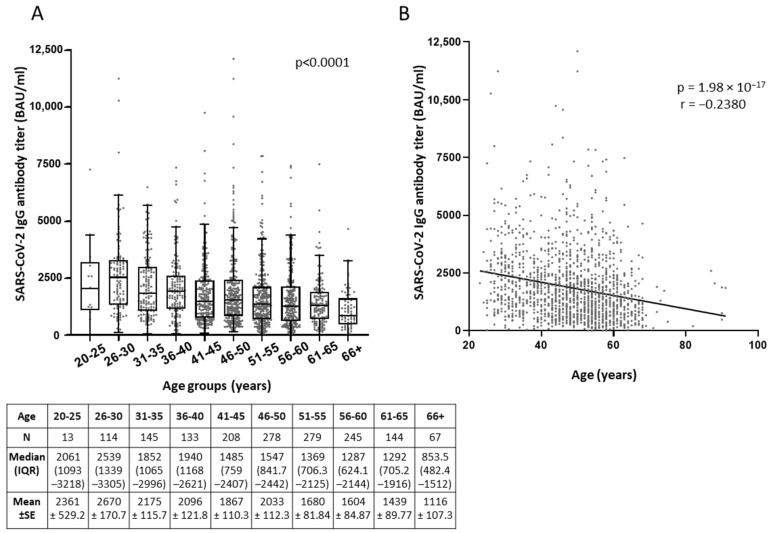
Correlation of anti-SARS-CoV-2 IgG antibody titers with the age of healthcare workers from the three hospitals Eginition, Evan/smos, and Kon/poulio (total population), after vaccination with the BNT162b2 mRNA vaccine. (**A**) Comparison of antibody titers (BAU/mL) among different age groups by One-Way ANOVA Kruskal-Wallis H test. Data are represented as XY scatter plot and box plots; line in the middle, median; box edges, 25th to 75th centiles; whiskers, range of values. Numeric results are presented in the table below. (**B**) XY scatter plot and fitted linear regression lines of anti-SARS-CoV-2 IgG titers versus age as continuous variable. Spearman’s correlation coefficient (r) and *p* values (*p*) were calculated.

**Figure 2:**
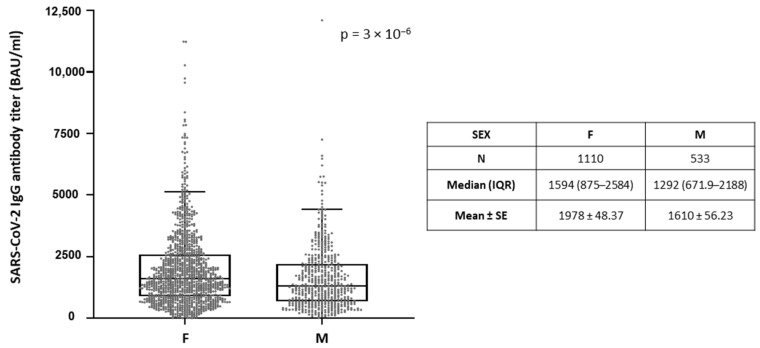
Comparison of antibody titers in men as compared to women. Data are represented as box plots; line in the middle, median; box edges, 25th to 75th centiles; whiskers, range of values. For the total population of vaccinees from Eginition, Evan/smos and Kon/poulio Hospitals, the significance of the difference between sexes was evaluated after calculation of *p* value with Mann-Whitney U test. Numeric results are presented in the table below.

The humoral response to vaccination was assessed in sera of vaccinees by quantitative determination of IgG antibodies to the SARS-CoV-2 spike S1 RBD, performing one of the two chemiluminescent microparticle sandwich immunoassays commonly used in Greece: SARS-CoV-2 IgG II Quant (Abbott) on the ARCHITECT i System (in the case of Eginition and Evangelismos Hospitals), or ADVIA Centaur SARS-CoV-2 IgG (COV2G, Siemens) on the Advia Centaur XP platform (Konstantopoulio Hospital), as defined by the policy of each hospital. All samples were processed according to the manufacturers’ procedures with the specified controls and calibrators. Samples with ≥50 arbitrary units (AUs)/mL in Abbott ARCHITECT IgG II Quant test and with ≥1.0 index values (defined as the ratio of the reading of the sample and the cut-off control) in Siemens ADVIA Centaur COV2G test, are considered positive for SARS-CoV-2 IgG, as suggested by the manufacturers. As reported by Abbott and Siemens, the results of their respective tests are equated to the binding antibody units (BAU)/mL of the first WHO International Standard for anti-SARS-CoV-2 immunoglobulins [28,29], as follows: Abbott assay: AU/mL × 0.142 = BAU/mL, Siemens assay: index values × 21.8 = BAU/mL. To combine the results for all healthcare worker vaccinees of the three hospitals (referred to as total population in Table 1 and Figures 1 and 2), we used the BAU/mL values that were calculated for the respective tests.

### 2.4. Statistics

Correlation of anti-spike protein antibody titers with vaccinees’ age at sample acquisition was determined by applying the non-parametric One-Way ANOVA Kruskal-Wallis H test (age categorized in groups) and Spearman’s correlation coefficient (age as continuous variable) after conducting the Shapiro–Wilk normality test. Mann–Whitney U test was performed to detect statistical differences for the anti-spike IgG titers between men and women. Fisher’s exact test was used to compare the ratio of males versus females in the groups of vaccinees. Statistical significance was assessed with the Prism (GraphPad Prism version 9.0.0 free trial, GraphPad Software Inc., San Diego, CA, USA) software.

## 3. Results

### 3.1. Subjects

Study population included a total of 1643 volunteer Greek healthcare workers (533 M/1110 F; median age: 49; interquartile range-IQR: 40–56) that were vaccinated with the COVID-19 BNT162b2 mRNA vaccine (Table 1). 242 individuals were from Eginition Hospital (96 M/146 F; median age: 51 years; IQR: 39–58 years), 999 from Evangelismos (Evan/smos) Hospital (315 M/684 F; median age: 48 years; IQR: 39–55 years), and 402 from Konstantopoulio (Kon/poulio) Hospital (122 M/280 F; median age: 51 years; IQR: 43–58 years).

### 3.2. Vaccine-Elicited Antibody Titers to SARS-CoV-2

The humoral response to vaccination was assessed in sera of vaccinees, 20–30 days after the second vaccine dose, by determining the IgG antibody titer against the SARS-CoV-2 spike S1 RBD. From the total population of vaccinees, 1636 (99.6%) had anti-SARS-CoV-2 IgG titers above the positivity threshold of the assay used. Specifically, from Eginition and Evangelismos Hospitals groups, 241 (99.6%) and 996 (99.7%) vaccinees, respectively, had ≥50 AU/mL, while from Konstantopoulio Hospital 399 (99.2%) individuals were reactive, with index values ≥1.0. The median (and IQR) values of the antibody titers were 8461 (4466–14,916) AU/mL for Eginition, 11,111 (5988–18,111) AU/mL for Evangelismos, and 67 (35–109) index values for Konstantopoulio, as well as 1483 (793.6–2431) BAU/mL for the total population of the three hospitals. The latter was calculated after equating the results of the respective tests (ARCHITECT IgG II Quant performed by Eginition and Evangelismos, and ADVIA Centaur COV2G used by Konstantopoulio) to the binding antibody units (BAU)/mL of the first WHO International Standard for anti-SARS-CoV-2 immunoglobulins and combining them (Table 1).

### 3.3. Age-Dependent and Gender-Dependent Antibody Responses against SARS-CoV-2 Vaccine

Interestingly, more robust anti-Spike immune responses were observed in the younger ages for all vaccinee groups studied (Figure 1, Figure S1). One-Way ANOVA Kruskal-Wallis H test showed that between the different age categories, difference in the median of antibody titers was statistically significant, with *p*-value < 0.0001 for the total population of vaccinees from the three hospitals (Eginition, Evangelismos, and Konstantopoulio) (Figure 1A, Figure S1A). Consistently, significant correlation of antibody titers with age as continuous variable was shown by Spearman’s correlation coefficient (r), which was −0.2380 (*p* = 1.98 × 10^−17^) for the total population (Figure 1B), as well as −0.252 (*p* = 1.13 × 10^−4^) for Eginition, −0.257 (*p* = 2.877 × 10^−16^) for Evangelismos, and −0.163 (*p* = 0.0014) for Konstantopoulio groups separately (Figure S1B). Moreover, the anti-SARS-CoV-2 IgG titers were slightly higher by 1.2-mean fold (*p* = 3 × 10^−6^)in the total female population of the three hospitals (median = 1594; IQR = 875–2584) as compared to males (median = 1292; IQR = 671.9–2188) (Figure 2), and by 1.3-mean fold (*p* = 0.0019) in the individuals of Eginition (median = 9778; IQR = 5257–15,623) and 1.2-mean fold (*p* = 0.001) in the individuals of Evan/smos (median = 11525; IQR = 6462–18,974) hospitals as compared to males (median = 7030; IQR = 3106–12,491 and median = 9778; IQR = 5260–15,865), respectively (Figure S2).

## 4. Discussion

Despite the high efficacy of the majority of currently approved vaccines, particularly at limiting the risk of clinical aggravation [30], accumulating data show a large heterogeneity in the immune response elicited after vaccination among different individuals [18,22,25,26,27]. The identification of important determinants of the post-COVID-19 mRNA BNT162b2 vaccine humoral response in the population is critical for designing more sufficient strategies for individuals predicted to be low vaccine responders, such as change of vaccine doses and booster intervals.

In agreement with previous reports [18,22,25,26,27], our data suggest a significant difference by age in the levels of anti-SARS-CoV-2 S1 RBD IgG antibodies after the second dose of BNT162b2 vaccine, with younger people producing higher antibody response in comparison to older people. Moreover, we found that women had a significantly higher humoral response compared to men after the second vaccine dose. This agrees with the recent studies of Salvagno et al. [18] and Terpos et al. [22], where the anti-Spike-RBD IgGs response was also observed to be more sustained in females than in males after vaccination with BNT162b2 vaccine. Another study, where the Euroimmun anti-SARS-CoV-2 S1 IgG ELISA assay was used to monitor humoral response to COVID-19 mRNA BNT162b2 vaccine, did not show any statistically significant correlation between the age and sex of the individuals and the immune response caused by the vaccine [23].

A critical challenge at present is to investigate the immune correlate(s) of protection from SARS-CoV-2 infection, including the antibody levels and the resulting neutralizing activity, the immunological memory, and cellular response elicited by the COVID-19 vaccine. In addition to the promotion of public health preventative measures, higher and/or more frequent vaccine doses could be planned for those populations that will be found to be less immunoreactive, thus ensuring adequate immune protection against SARS-CoV-2 in the community, as also recently proposed by other studies [18,31]. In this context, the efficacy of a third dose in people aged 65 to 85 years, who have received their first two doses of BNT162b2 in the phase III trial, is being currently evaluated by Pfizer [32].

## 5. Conclusions

The results of this study, aiming at monitoring anti-SARS-CoV-2 RBD IgG antibodies response after Pfizer BNT162b2 mRNA COVID-19 vaccination, reveal that the vaccine is particularly effective in producing high anti-SARS-CoV-2 IgGs in healthy individuals that have no history or laboratory evidence of prior SARS-CoV-2 infection and do not receive immunosuppressive therapy. Interestingly, this humoral immune response is age-dependent and gender-dependent. Future studies should be performed to investigate whether other variables, such as the presence of comorbidities, body mass index, and physical activity, may affect humoral response to BNT162b2 mRNA vaccination.

## Figures and Tables

**Table 1:** Demographic data and anti-SARs-CoV-2 IgG titers of healthcare worker vaccinees included in the study.

		Eginition	Evan/Smos	Kon/Poulio	Total
**Vaccines Νο**		242	999	402	1643
**Median IgG** **titers (IQR)**		8461 (4466–14,916) AU/mL	11,111 (5988–18,111) AU/mL	67 (35–109) index values	1483 (793.6–2431) BAU/mL
**Median age in years (IQR)**		51 (39–58)	48 (39–55)	51 (43–58)	49 (40–56)
**Age group**	20–25	1	8	4	13
	26–30	10	92	12	114
	31–35	34	94	17	145
	36–40	18	85	30	133
	41–45	24	125	59	208
	46–50	27	180	71	278
	51–55	45	162	72	279
	56–60	41	130	74	245
	61–65	21	78	45	144
	66+	10	45	12	67
	NR	11	0	6	17
**Sex**	Female	146	684	280	1110
	Male	96	315	122	533
***p*-value**		0.0163 ^a^	0.7022 ^b^		

NR: Data not recorded; IQR: inter-quartile range; ^a,b^ Fisher’s exact test for the ratios. No females/No males between Eginition and Kon/poulio (a) or Evan/smos and Kon/poulio (b).

## Data Availability

All relevant data are within the manuscript and its Supporting Information files.

## Supplementary Materials

The following are available online at https://www.mdpi.com/article/10.3390/microorganisms9081725/s1, Figure S1: Correlation of anti-SARS-CoV-2 IgG antibody titers with the age of healthcare workers from each of the three hospitals Eginition, Evan/smos, or Kon/poulio, after vaccination with the BNT162b2 mRNA vaccine, Figure S2: Comparison of antibody titers in men as compared to women from each hospital.
