# Supplementary material for: Impact of Age and Sex on Antibody Response Following the Second Dose of COVID-19 BNT162b2 mRNA Vaccine in Greek Healthcare Workers
Source: Microorganisms. 2021 Aug 13;9(8):1725. doi: 10.3390/microorganisms9081725 (PMC8401044; doi:10.3390/microorganisms9081725)
Supplement: Supplementary file 1 [file microorganisms-09-01725-s001.zip › Supplementary _figure_legends.pdf]

Supplementary figure legends

Figure S1. Correlation of anti-SARS-CoV-2 IgG antibody titers with the age of healthcare workers from each of the three hospitals Eginition, Evan/smos or Kon/poulio, after vaccination with the BNT162b2 mRNA vaccine. (**A**) Comparison of antibody titers (AU/ml or index values) among different age groups by One-Way ANOVA Kruskal-Wallis H test. Data are represented as XY scatter plot and box plots; line in the middle, median; box edges, 25th to 75th centiles; whiskers, range of values. (**B**) XY scatter plot and fitted linear regression lines of anti-SARS-CoV-2 IgG titers versus age as continuous variable. Spearman's correlation coefficient (r) and p values (p) were calculated.

Figure S2. Comparison of antibody titers in men as compared to women from each hospital. Data are represented as box plots; line in the middle, median; box edges, 25th to 75th centiles; whiskers, range of values. For the vaccinees from each of the three hospitals Eginition, Evan/smos or Kon/poulio, the significance of the difference between sexes was evaluated by calculating p values with Mann-Whitney U test.
