# Supplementary figures and images for: Impact of Age and Sex on Antibody Response Following the Second Dose of COVID-19 BNT162b2 mRNA Vaccine in Greek Healthcare Workers
Source: Microorganisms. 2021 Aug 13;9(8):1725. doi: 10.3390/microorganisms9081725 (PMC8401044; doi:10.3390/microorganisms9081725)

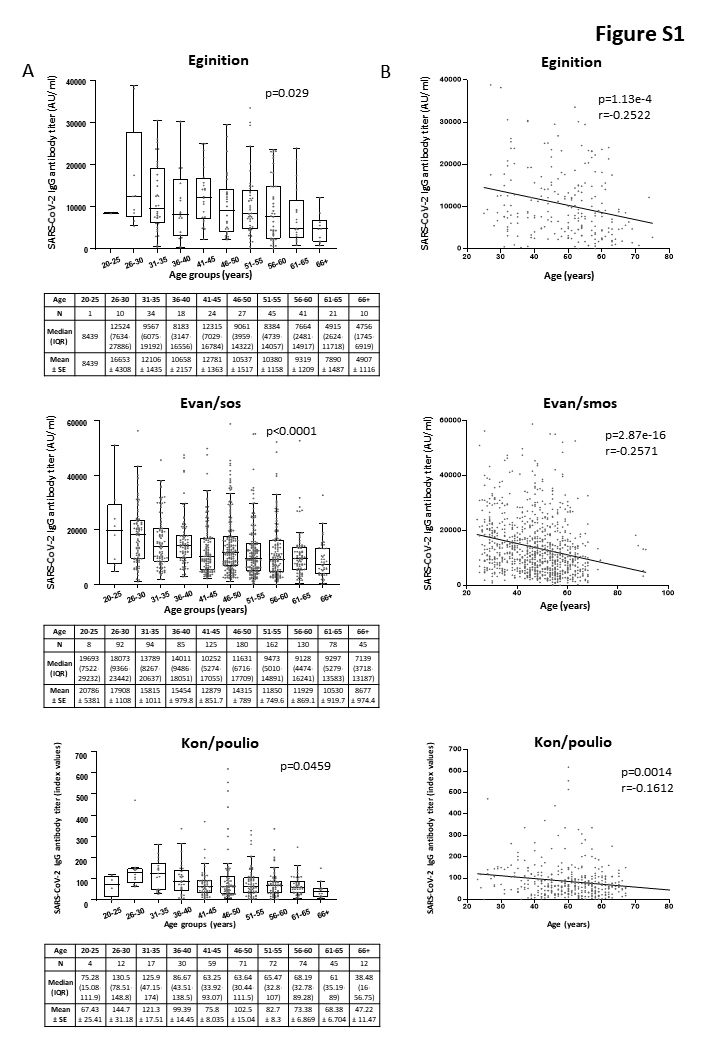

Supplement: Supplementary file 1 [file microorganisms-09-01725-s001.zip › Figure_S1.TIF]

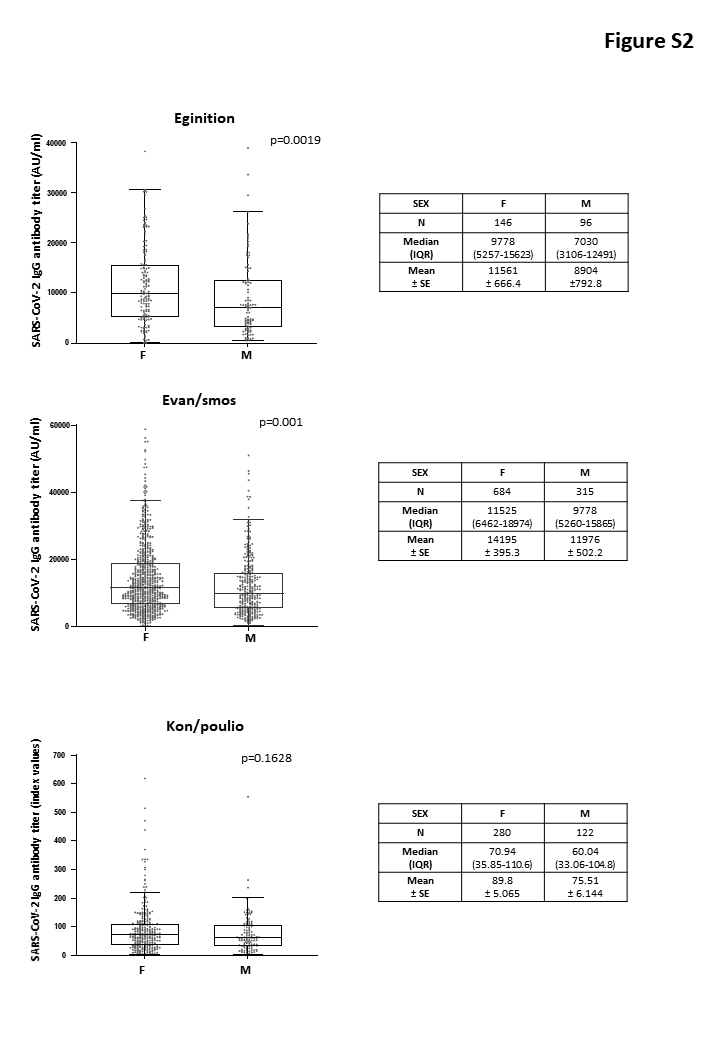

Supplement: Supplementary file 1 [file microorganisms-09-01725-s001.zip › Figure_S2.TIF]
